# Modification of a Hydrophobic Layer by a Point Mutation in Syntaxin 1A Regulates the Rate of Synaptic Vesicle Fusion 

**DOI:** 10.1371/journal.pbio.0050072

**Published:** 2007-03-06

**Authors:** Robert D Lagow, Hong Bao, Evan N Cohen, Richard W Daniels, Aleksej Zuzek, Wade H Williams, Gregory T Macleod, R. Bryan Sutton, Bing Zhang

**Affiliations:** 1 Section of Neurobiology and Institute for Neuroscience, University of Texas at Austin, Austin, Texas, United States of America; 2 Department of Neuroscience and Cell Biology, University of Texas Medical Branch, Galveston, Texas, United States of America; Princeton University, United States of America

## Abstract

Both constitutive secretion and Ca^2+^-regulated exocytosis require the assembly of the soluble *N*-ethylmaleimide–sensitive factor attachment protein receptor (SNARE) complexes. At present, little is known about how the SNARE complexes mediating these two distinct pathways differ in structure. Using the *Drosophila* neuromuscular synapse as a model, we show that a mutation modifying a hydrophobic layer in syntaxin 1A regulates the rate of vesicle fusion. Syntaxin 1A molecules share a highly conserved threonine in the C-terminal +7 layer near the transmembrane domain. Mutation of this threonine to isoleucine results in a structural change that more closely resembles those found in syntaxins ascribed to the constitutive secretory pathway. Flies carrying the I254 mutant protein have increased levels of SNARE complexes and dramatically enhanced rate of both constitutive and evoked vesicle fusion. In contrast, overexpression of the T254 wild-type protein in neurons reduces vesicle fusion only in the I254 mutant background. These results are consistent with molecular dynamics simulations of the SNARE core complex, suggesting that T254 serves as an internal brake to dampen SNARE zippering and impede vesicle fusion, whereas I254 favors fusion by enhancing intermolecular interaction within the SNARE core complex.

## Author Summary

Most living cells constantly renew their membrane compositions and frequently communicate with neighboring cells by delivering cargo molecules from small vesicles. A key step in cargo delivery requires the fusion of the vesicle membrane with the target membrane mediated by SNARE proteins. In most cellular compartments, fusion occurs constitutively, requiring little participation of other molecules. In other cellular compartments, such as synapses in the nervous system, vesicle fusion is predominantly triggered by intracellular calcium ions. At present, constitutive and regulated fusion modes are not well understood.

In this study, we found that a mutant SNARE protein, syntaxin at the synapse, contained a building block commonly conserved for syntaxins functioning along constitutive secretory pathways. Further, our modeling predicted that the mutant syntaxin could form a tightly packed SNARE bundle closely resembling that found in the endosome, but differing from the relatively loosely packed bundle found at the wild-type synapse. Our experimental data support the hypothesis that the mutant syntaxin lowered the energy barrier for vesicle fusion by tightening the SNARE bundle. These findings reveal a novel, intrinsic structural feature of the SNARE complex that regulates vesicle fusion rate at different cellular compartments.

## Abstract 3

A syntaxin 1A threonine to isoleucine mutation is found to enhance SNARE complex formation and vesicle fusion. This structural change results in a syntaxin that resembles those in constitutive secretory pathways.

## Introduction

**Figure 1:**
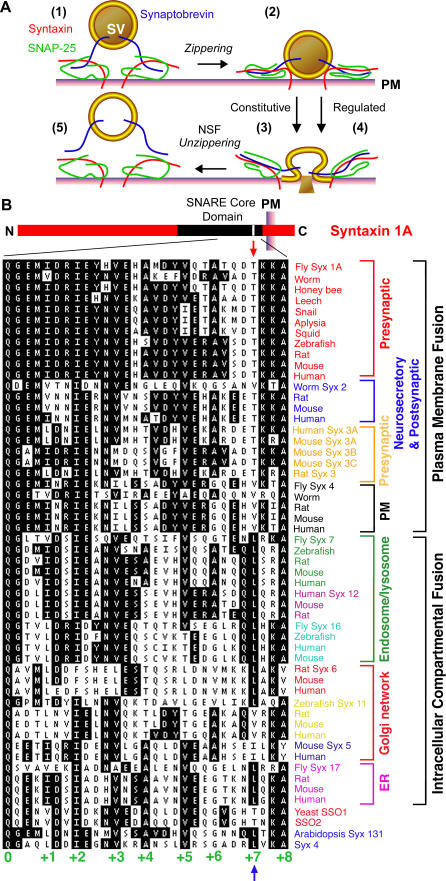
Conservation and Divergence of Threonine 254 among Different Syntaxin Orthologs (A) Proposed model of SNARE complex assembly and disassembly in a synaptic vesicle cycle (adapted from [5]). (1) Synaptobrevin forms a partial *trans* SNARE complex with syntaxin 1A and SNAP-25. (2) By zippering in an N- to C-termini direction, the SNARE proteins form a *trans* complex and bring the synaptic vesicle close to the plasma membrane. SNARE-mediated synaptic vesicle exocytosis occurs either spontaneously (3) or evoked by Ca^2+^ (4). (5) *cis* SNARE complexes are thought to be disassembled by NSF ATPase prior to vesicle recycling. ER, endoplasmic reticulum; PM, plasma membrane; SV, synaptic vesicle. (B) Alignment of amino acids (aa) around position T254 in the *Drosophila* syntaxin 1A or equivalent residues in syntaxin orthologs from a variety of animals, yeast, and the plant *Arabidopsis*. The top panel shows a cartoon of syntaxin 1A and the region of the alignment. Syntaxins are organized as “plasma membrane” or “intracellular compartments” according to their cellular distributions. With the exception of syntaxin 4, most plasma membrane syntaxins are known to function in presynaptic terminals or neurosecretory cells for Ca^2+^-regulated exocytosis. Note that T254 is highly conserved among “presynaptic” syntaxin 1A, 2, and 3A molecules. We call all other syntaxin orthologs shown here “constitutive” syntaxins because they are used for constitutive secretion on the plasma membrane (PM) and intracellular compartments, such as the endosome and the lysosome, the *cis* and *trans* Golgi network (Golgi network), and endoplasmic reticulum (ER) [1]. The yeast plasma membrane syntaxin orthologs SSO1 and SSO2, and syntaxins 4 and 131 from *Arabidopsis* are also shown here. (A more complete alignment can be see in Figure S1.) Unlike the synaptic syntaxins, syntaxin 4 and most syntaxin 11s have a valine (V) at the 254 equivalent position, syntaxins 6, 7, 12, 16, and 17 a leucine (L), and syntaxin 5 an isoleucine (I). The isoleucine found in the *syx^3–69^* mutant resembles some of the wild-type syntaxin orthologs used for constitutive secretion. The core complex layers from 0 to +8 are identified at the bottom. The aa sequence was obtained from the NIH's National Center for Biotechnology Information (NCBI; http://www.ncbi.nlm.nih.gov) and aligned using the software DNAStar.

Soluble *N*-ethylmaleimide–sensitive factor (NSF) attachment protein receptor (SNARE) proteins are thought to mediate vesicle fusion in all eukaryotes [1–4]. In nerve terminals, there are two target-SNAREs (t-SNAREs, also called Q-SNAREs), syntaxin 1A and synaptosome-associated protein-25 kDa (SNAP-25) on the plasma membrane, and one vesicle-associated SNARE (v-SNARE, also called R-SNARE), synaptobrevin 2 on synaptic vesicles [2]. Prior to exocytosis, the t- and v-SNAREs are thought to form a *trans* complex composed of a four-stranded helical bundle with one helix each from syntaxin and synaptobrevin and two helices contributed by SNAP-25 [5–9] (Figure 1A). As vesicles undergo fusion, the SNARE complex rearranges from a *trans* to a *cis* configuration such that all the SNARE proteins are localized to one membrane. The *cis* complex is then thought to be rapidly disrupted by the ATPase NSF [5,10–12], allowing the v-SNARE to be recycled into synaptic vesicles [13]. Although the specific mechanism of vesicle fusion is still in debate, it is now widely accepted that the formation of this four-helix bundle is essential for the fusion of the vesicle phospholipid bilayer with the plasma membrane phospholipid bilayer [3].

Vesicle fusion can be constitutive or triggered by calcium ion (Ca^2+^) [14]. In the latter case, the putative Ca^2+^ sensor synaptotagmin I plays a critical role [2,3]. Constitutive vesicle fusion differs from regulated secretion in that it is relatively less dependent on intracellular Ca^2+^. This has been demonstrated in reconstituted secretory cells [15] and at synapses, including mammalian [16,17] and invertebrate nerve terminals [18]. In these preparations, removal of extracellular Ca^2+^ or reduction of intraterminal [Ca^2+^] by Ca^2+^ chelators does not stop spontaneous vesicle fusion. At the *Drosophila* larval neuromuscular junction (NMJ), Ca^2+^-free saline containing ethylene glycol tetraacetic acid (EGTA) does not alter the rate of spontaneous release [19]. These observations collectively suggest that spontaneous vesicle fusion can occur even when intracellular [Ca^2+^] is reduced. This implies that a mechanism exists to overcome the energy barrier for vesicle fusion at low-Ca^2+^ conditions. Because SNARE complexes also mediate vesicle fusion along the constitutive secretory pathway [1,20], it is conceivable that this mechanism lies within the different structural and/or biochemical properties of SNARE complexes used for constitutive secretion and Ca^2+^-regulated exocytosis.

The synapse offers an ideal site to test this hypothesis because both forms of secretion co-exist and the SNARE proteins involved in the process are well studied. Furthermore, vesicle fusion can be readily detected at single-vesicle levels using electrophysiology [14]. In this study, we focused on a point mutation, T254I in syntaxin 1A, located at the +7 layer of the SNARE core complex [6], and its role in SNARE complex assembly and synaptic transmission in *Drosophila*. In an earlier study [21], it was demonstrated that this mutation *(syx^3–69^)* completely abolished the assembly of the SNARE complex at the restrictive temperature. Consequently, synaptic transmission was fully blocked and the fly paralyzed. Along with previous genetic deletion or mutation studies [22–24], these results provided important in vivo evidence that SNARE complex assembly was essential for synaptic vesicle fusion. However, our re-investigation of the *syx^3–69^* mutant shows that the T254I mutation blocks neither the assembly of the SNARE complex nor synaptic transmission at the restrictive temperature. Instead, we find that the T254I mutation promotes the formation of the SNARE complex as well as vesicle fusion at permissive temperatures. These findings are consistent with a molecular model of the SNARE complex, suggesting that the T254I mutation causes a structural change of the +7 layer so that the mutant layer more closely resembles those found along constitutive secretory pathways. By enhancing the hydrophobic core of the molecule in the vicinity of layer +7, towards the C-terminal transmembrane helix, the mutant SNARE complex favors “constitutive-like” vesicle secretion either by increasing intermolecular interactions among the SNARE bundles or by stimulating vesicle docking and/or priming. These results suggest an evolutionarily conserved mechanism intrinsic to the structure of SNARE complexes that could act as a molecular switch to regulate the rate of vesicle fusion.

## Results

### The *syx^3–69^* Mutation Does Not Block Synaptic Transmission or SNARE Complex Assembly at Restrictive Temperatures

Syntaxin 1A is a critical component of the SNARE complex and is thought to be essential for synaptic vesicle fusion [1–3,24]. A previous study showed that mutation of threonine (T) to isoleucine (I) at position 254 in the *Drosophila* syntaxin 1A was sufficient to abolish the assembly of SNARE complexes at restrictive temperatures [21]. However, this conclusion is questionable if we take into consideration the conservation and divergence of residues at this position among different syntaxins. Our sequence analysis shows that, with the exception of syntaxin 4, most syntaxins found at the plasma membrane have a highly conserved T254 residue at the +7 layer (Figure 1B). Notably, the T254-containing syntaxins, such as syntaxin 1, 2, and 3, are typically used for regulated vesicle fusion at either synapses or neurosecretory cells in a diverse range of animal species [25–27]. In contrast, syntaxins involved in most constitutive secretion pathways in both animals and plants have one of the following amino acids at their equivalent positions: isoleucine, leucine (L), or valine (V) (Figure 1B; see also Figure S1). Valine, leucine, and isoleucine are similar in that they are hydrophobic, branch-chained amino acids. Therefore, this substitution of the residue at position 254 among syntaxins in the constitutive pathways is highly conserved throughout evolution. There are a few exceptions to this generalization. The yeast plasma membrane syntaxin orthologs have a threonine at the equivalent position (Figures 1B and S1). Furthermore, T254-containing syntaxins could also function in non-synaptic secretions, such as syntaxin 2 in postsynaptic membrane trafficking [26] and *Drosophila* syntaxin 1A in cuticle secretion [23,28]. Nonetheless, the overall feature emerging from our analysis is that syntaxins with conserved isoleucine at the +7 layer appear to be selectively involved in regulated secretion at synapses or neurosecretory cells.

**Figure 2:**
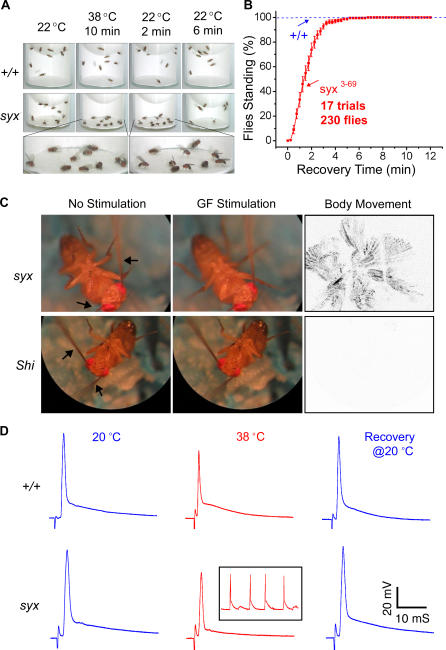
Behavioral and Electrophysiological Analyses Reveal That Synaptic Transmission Is Not Blocked in the *syx^3–69^* Mutant Fly at Restrictive Temperatures (A and B) Temperature-sensitive paralysis and recovery of the *syx^3–69^* mutant fly. (A) shows the still image of both wild type (+/+) and the *syx^3–69^* mutant before, during, and after exposure to the restrictive temperature (38 °C). Although the wild-type flies are not paralyzed at 38 °C, the *syx^3–69^* mutant flies are. However, the *syx^3–69^* flies recover rapidly to standing position within 2–3 min once returned to the permissive temperature. The quantification of the recovery kinetics is shown in (B). Error bars in this and all other figures indicate the standard errors. (C) The paralyzed *syx^3–69^* mutant flies remain capable of responding to stimuli via the polysynaptic giant fiber (GF) pathway. The flies are anchored on a glass slide upside down with modeling clay while a stimulating electrode is inserted into one of the compound eyes (arrows). The *syx^3–69^* fly constantly shakes its legs, head, and abdomen while paralyzed at 38 °C. In response to electrical stimulation of the giant fiber neuron, the mutant fly extends its legs phase-locked with each stimulus. However, the *Shi^ts1^* fly is completely paralyzed and does not respond to the stimuli at the same restrictive temperature. The right-most panels summarize the cumulative spontaneous and electrical stimulation–evoked movements of legs in *syx^3–69^* flies and the lack of leg movement in *Shi^ts1^* flies. These behavioral observations strongly indicate that exposing the *syx^3–69^* fly to 38 °C does not block synaptic transmission. See also Videos S4 and S5. (D) Recordings from indirect flight muscles confirm that synaptic transmission is not blocked in *syx^3–69^* flies at the restrictive temperature. Action potentials in DLMs driven by polysynaptic stimuli along the giant fiber pathway remain the same in the *syx^3–69^* mutant fly as in the wild-type control fly before, during, and after exposure to the restrictive temperature. Synaptic-induced high-frequency action potentials are often observed in both the wild type and the *syx^3–69^* mutant (unpublished data). These high-frequency action potentials also occur spontaneously in the mutant. (An example is shown in the inset box.)

It is particularly interesting to note that the T254I mutation found in the *syx^3–69^* mutant approximates a reversion to a residue of wild-type syntaxins found in the constitutive secretory pathway. Notably, syntaxin 5 isoforms place an isoleucine at the site equivalent to position 254. Syntaxin 5 clearly functions in mammal *cis* Golgi networks at normal body temperature, similar to the temperature at which the *syx^3–69^* mutant is reported to lose the ability to form SNARE complexes [21]. This prompted us to reconsider whether the T254I mutant syntaxin 1A indeed ceases to function at restrictive temperatures. To this end, we thoroughly re-examined the behavior, synaptic transmission, and SNARE complex formation of the *syx^3–69^* mutant fly at elevated temperatures. Our tests showed that *syx^3–69^* mutant flies were rapidly paralyzed at 38 °C and recovered within 3 min when returned to permissive temperature after a 20-min period of paralysis (Figure 2A and 2B). The paralysis and recovery rates were identical to those shown previously [21]. However, different from the previous observations, we noted that the *syx^3–69^* mutant fly was paralyzed, but not motionless: the flies constantly shook their legs and abdomens during the period of paralysis at 38 °C (compare Video S1 with Video S2). We used a wild-type fly *(Canton-S [CS])* and two other temperature-sensitive paralytic flies, *Shibire^ts1^ (Shi^ts1^)* and *paralytic^ts1^ (para^ts1^)* as controls. As expected, *Shi^ts1^* and *para^ts1^* flies were completely paralyzed due either to a block of synaptic vesicle recycling [29] or a failure of action potential propagation [30], respectively, and did not exhibit the shaking seen in the *syx^3–69^* mutant. Upon returning to room temperature, *Shi^ts1^*, *para^ts1^,* and *syx^3–69^* flies all resumed their normal activities (Video S3).

These behavioral observations suggest that synaptic transmission persists in *syx^3–69^* flies at the restrictive temperature. To further test this idea, we examined leg movement upon the activation of the giant fiber pathway in adult flies [31]. We stimulated the giant fiber neurons located in the head and observed the movement of fly legs (the body and wings were anchored with wax on a slide). Repetitive and phase-locked leg shaking was readily observed in *syx^3–69^* flies at both the permissive temperature (20 °C; unpublished data) and restrictive temperature (38 °C) following each stimulus of the giant fiber neurons (see Video S4). In contrast, *Shi^ts1^* flies moved their legs in response to each stimulus only at the permissive temperature (20 °C; unpublished data), but not at the restrictive temperature (Video S5). Figure 2C (rightmost panels) summarizes the spontaneous and electrical stimulation–evoked leg movement in *syx^3–69^* flies and the lack of such movement in *Shi^ts1^* flies at restrictive temperatures.

The persistence of synaptically evoked leg movements at the restrictive temperature suggests that synaptic transmission remains intact through multiple synapses (an electrical synapse and two chemical synapses) along the giant fiber pathway [31]. To directly measure synaptic transmission, we next recorded the synaptic response of the dorsal longitudinal indirect flight muscles (DLMs) from *syx^3–69^* flies maintained at 38 °C. Our results show that evoked synaptic transmission and the resulting action potential persisted at 38 °C (*n* = 6; Figure 2D). During the course of these experiments, we noted that intracellular electrodes were often dislodged from DLMs only from *syx^3–69^* flies, and there was a high incidence of spontaneous action potentials in the mutant DLMs (Figure 2D, inset). In contrast, *Shi^ts1^* flies completely lost synaptic transmission upon activation of the giant fiber neuron at restrictive temperatures [32] (unpublished data). Hence, synaptic transmission is not blocked at restrictive temperatures in *syx^3–69^* flies. As shown below, it is likely that paralysis of the *syx^3–69^* mutant is caused by excessive or uncoordinated release of transmitter, rather than a complete block of exocytosis as suggested previously [21].

**Figure 3:**
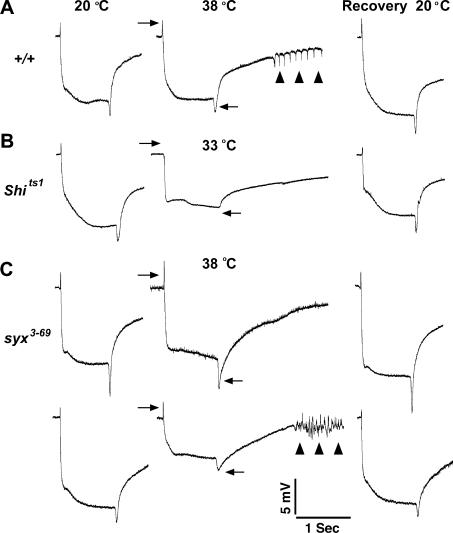
ERG Recordings Show That Synaptic Transmission Is Not Blocked in the *syx^3–69^* Mutant Fly at Restrictive Temperatures (A) ERGs are obtained from the wild-type fly at the permissive temperature (20 °C), during the restrictive temperature (38 °C), and during recovery at 20 °C following a brief white-light stimulation of the compound eye. The spikes before and following the sustained photoreceptor potential are called “on” and “off” transient potentials (arrows), respectively. They are thought to reflect synaptic transmission from the photoreceptor to downstream interneurons. Note that these transient potentials are not significantly affected at 38 °C. The extracellular recording electrode also detects light-induced high-frequency action potentials (arrowheads), which normally result in startle escape. (B) The “on” and “off” transient potentials are absent in *Shi^ts1^* flies exposed at 33 °C, consistent with a conditional block of vesicle recycling. (C) Under the same experimental conditions, the “on” and “off” transient potentials in the *syx^3–69^* mutant fly remain essentially similar to those observed in the wild-type fly. Even though the amplitude of photoreceptor potentials is reduced and the duration of recovery is prolonged, synaptic transmission is not blocked at 38 °C in both the wild-type and the mutant fly. Additionally, the mutant fly also displays light-induced high-frequency action potentials (arrowheads), even though it is paralyzed.

Consistent with the observation that synaptic transmission persists along the giant fiber pathway, light-induced “on” and “off” transient potentials of electroretinograms (ERGs) were not blocked by exposure of the *syx^3–69^* fly to the restrictive temperature (Figure 3). These transients are thought to reflect synaptic transmission from photoreceptors to downstream interneurons in the retina [33]. The control fly, *Shi^ts1^,* lost its transient potentials at 33 °C, consistent with a depletion of the vesicle pool [21,29,32] (Figure 3B). However, the findings from the *syx^3–69^* fly differ from those reported earlier [21], which showed that the restrictive temperature reversibly blocked these transients. In our experiments, we carefully monitored the temperature of the *syx^3–69^* fly by placing a temperature probe adjacent to the experimental fly. Additionally, we mounted another *syx^3–69^* fly beside the experimental fly so that we could observe the paralysis during the exposure at 38 °C and the recovery afterward. In a total of eight experiments, we never saw a loss of these transient potentials. In fact, our results showed that the “on” transient potential was slightly increased in amplitude at 38 °C (see Figure 3C). Additionally, we also observed spontaneous and light-induced high-frequency “bursting” activities typically indicative of enhanced neuronal activity in both the wild-type and the *syx^3–69^* flies (see arrowheads in Figure 3A and 3C; see also [34]). Hyperactivity of the thoracic ganglion was also observed independently by Dr. Bruno van Swinderen's laboratory when *syx^3–69^* flies were exposed to the restrictive temperature (B. van Swinderen, personal communication).

**Figure 4:**
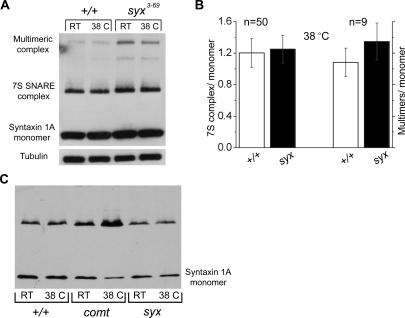
The SNARE Complexes Remain in *syx^3–69^* Mutant Flies at the Restrictive Temperature (A and B) The SDS-resistant complex is not obviously affected in homozygous *syx^3–69^* mutant flies at restrictive temperatures. A representative Western blot shows the syntaxin 1A monomer, the 7S SNARE complex, and the multimeric complex obtained from heads of the wild type (+/+) and the *syx^3–69^* mutant (A). A control for total protein loaded is illustrated by the intensity of tubulin (bottom). Histograms of ratios of the 7S and multimeric complexes to monomer in wild-type (+/+) and *syx^3–69^* mutant flies are shown in (B). Unless specifically noted, the complex-to-monomer ratio is normalized to that of the wild type at room temperature in this and other SNARE complex histograms. The SNARE complex was extracted from flies either at room temperature (∼22 °C) or after exposure to 38 °C for 20 min, as described above [21]. (C) An example of Western blots showing the SNARE complex in the wild-type (+/+), *comatose (comt^tp7^),* and *syx^3–69^* mutant flies. The SNARE complex accumulates in the *comt^tp7^* mutant, likely as a result of a block of the NSF ATPase activity at the restrictive temperature. Note that even though relatively less protein was loaded in the *syx^3–69^* lanes (as judged by the intensity of the syntaxin band), the 7S complex remains in paralyzed *syx^3–69^* flies.

Taken together, both our behavioral tests and electrophysiological analyses support the notion that synaptic transmission is not blocked in *syx^3–69^* mutants at restrictive temperatures. These results further suggest that the formation of the SNARE complex is not abolished in *syx^3–69^* mutants at the restrictive temperature. To test this hypothesis, we measured the level of SNARE complexes using the methods described previously [11,21]. We first established the “linear” range that allows optimal detection of changes in the sodium dodecyl sulfate (SDS)-resistant SNARE complex (Figure S2) and then measured the level of SNARE complexes in *syx^3–69^* mutants. Our results showed that the amount of the 7S SNARE complex and high molecular weight SNARE multimers (or oligomers) remained at wild-type levels in *syx^3–69^* mutant flies at the restrictive temperature (Figure 4A and 4B). The *syx^3–69^* mutant fly was exposed to 38 °C for 20 min prior to rapid freezing with liquid nitrogen and extraction of the SDS-resistant SNARE complex, as described previously [21]. In 50 separate experiments, we consistently observed the SNARE complex. This result has also been independently noted in Dr. Leo Pallanck's laboratory (L. Pallanck, personal communication). In a number of experiments, we also included the *comatose (comt)* mutant in our Western analysis and detected a consistent accumulation of the SNARE complex (Figure 4C), which is thought to be caused by dysfunction of NSF at restrictive temperatures [10–12]. Taken together, all our observations show that the T254I mutation in syntaxin 1A does not block SNARE complex formation nor does it block synaptic transmission at restrictive temperatures. Because our results differ markedly from those reported earlier [21], we sought to confirm whether the mutant fly we studied indeed carried the T254I mutation as shown in the *syx^3–69^* mutant. Sequencing confirmed that there is a single base change from ACC to ATC in the open reading frame of syntaxin 1A (see Figure S3). Furthermore, we were able to rescue the paralysis (unpublished data) and electrophysiological defects by neuronal expression of the wild-type syntaxin 1A in the *syx^3–69^* mutant background (see below). These results leave little doubt that the phenotype we study here is specifically caused by the T254I mutation in *syx^3–69^* mutant flies.

### Structural Modeling Suggests That the T254I Mutation Tightens SNARE Complexes

**Figure 5:**
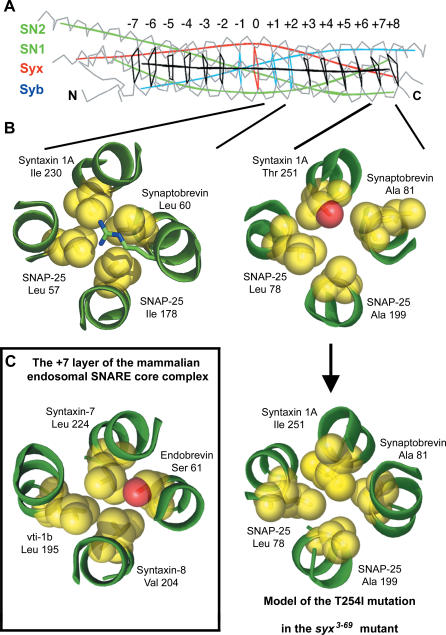
Structural Modeling Suggests That the T254I Mutation in Syntaxin 1A Increases Direct Molecular Interactions within the +7 Layer (A) The core complex layers of the synaptic SNARE complex (1SFFC), consisting of two α-helical bundles from SNAP-25 (SN1 and SN2) and one bundle each from syntaxin 1A (Syx) and synaptobrevin (Syb), are shown (adapted from [6]). Although initially obtained as *cis* complexes with truncated SNAREs [6], these layers of the core complex are most likely found in pre-fusion *trans* SNARE complexes. (B) Crystal structures of +1 and +7 layers of the synaptic core complex (1SFC [6]) show tightly and loosely packed bundles, respectively. Note the void space within the +7 layer. Our structural modeling shows that the mutation of the hydrophilic threonine at position 251 (which is equivalent to position 254 in *Drosophila* syntaxin 1A) to a hydrophobic isoleucine results in a relatively tightly packed +7 layer. This may allow direct molecular interactions between syntaxin 1A with its neighboring bundles from SNAP-25 and synaptobrevin. It is hypothesized that the T254I mutation in *syx^3–69^* stimulates vesicle fusion by lowering the energy barrier for zippering of the SNARE complex. (C) Representative +7 layer abstracted from the crystal structure of the endosomal SNARE (1GL2 [20]). Note that this layer is tightly packed and similar to the T251I mutant layer. Given the evolutionary conservation of hydrophobic residues at the +7 layer among “constitutive” syntaxin orthologs (Figure 1B), this structural resemblance suggests that the T254I mutant syntaxin 1A may function as a constitutive syntaxin to promote vesicle fusion.

To account for the hyperactivity observed in *syx^3–69^* flies, we next examined whether the T254I mutation in syntaxin 1A has any effect on SNARE assembly and synaptic function at permissive temperatures. Upon examination of the available crystal structures of SNARE core complexes [6], we found that many of the central layers are tightly packed with hydrophobic residues contained within the four helical bundles. An example of this tight packing in the +1 layer of the synaptic SNARE core complex is illustrated in Figure 5A and 5B. The interactions of Leu57 and Ile178 from SNAP-25, Ile230 from syntaxin 1A, and Leu60 from synaptobrevin form square-planar geometry typical of the leucine zipper motif. In contrast, the +7 layer containing the wild-type syntaxin 1A is relatively loosely packed due to the presence of a conserved polar threonine residue at position 251 (equivalent to position 254 in *Drosophila* syntaxin 1A) [6,7,21], which packs against more hydrophobic partners. Results of examination of homologous neuronal SNARE syntaxin proteins implied a similar loosely packed configuration in this layer [7]. Interestingly, the homologous layer of the endosomal SNARE X-ray structure (1GL2) [20] shows more reliance on hydrophobic, branched-chain amino acids, than the synaptic SNARE (Figure 5C). The resulting interaction may contribute more hydrophobic stability of the zippered endosomal complex relative to the wild-type synaptic SNARE complex. We therefore propose that the tightened +7 layer in the SNARE complex containing the T254I mutant syntaxin 1A may mimic the function of the endosomal complex.

Our modeling results do not support the observation that the T254I mutation debilitates SNARE complex assembly, as previously reported [21]. To further verify this, we conducted molecular dynamics simulations of the SNARE complex in a water bath at 300 K for 5 ns using GROMACS [35,36]. After equilibration was achieved, there was no gross difference between the interactions of wild-type SNARE components and the mutant SNARE components. Also, the wild-type SNARE structure shows some degree of “fraying” at the termini of the complex [6]. Although this fraying effect is probably not physiologically relevant, per se, it does illustrate the looser packing of residues at the periphery of the wild-type complex. In our simulations, one would expect a destabilization of the termini (increase in fraying) if this mutation were indeed unstable; however, none was observed. Our simulation shows that the T254I mutation does not destabilize the complex, nor does it obviously increase the fraying at the terminus relative to wild-type.

Based on this structural analysis and modeling, we predict that the T254I mutation facilitates the formation or stability of the SNARE complex by enhancing intermolecular hydrophobic interactions among the four SNARE α-helices. Because this layer is near the C-terminal of the SNARE core complex, a tighter zippering of the SNARE complex may make fusion more probable by lowering the energy barrier for fusion and thereby partially abrogate the Ca^2+^-dependence of exocytosis. The mutant protein could also promote vesicle fusion by enhancing vesicle docking/priming. In other words, the T254I mutation may increase the rate of spontaneous release, turning the synapse into a constitutive secretion site. Alternatively, the T254I mutation could stabilize the *cis* SNARE complex such that it impedes vesicle recycling and ultimately reduces exocytosis upon repetitive nerve stimulation.

### The Assembly of SNARE Complexes Is Enhanced in the *syx^3–69^* Mutant at the Permissive Temperature

**Figure 6:**
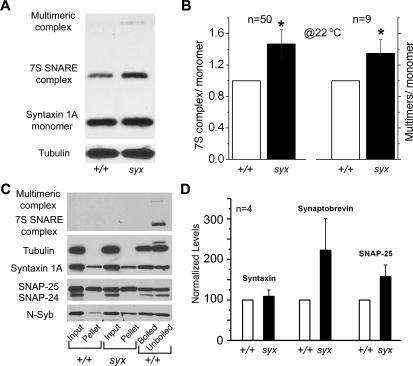
The Assembly of SDS-Resistant SNARE Complexes Is Increased in *syx^3–69^* Mutant Flies at Permissive Temperatures (A and B) The amount of SDS-resistant 7S complex as well as the multimeric complex is significantly increased in homozygous *syx^3–69^* mutant flies at 22 °C. A representative Western blot shows the syntaxin 1A monomer, the 7S SNARE complex, and the multimeric complex obtained from heads of the wild type (+/+) and the mutant (*syx* [A]). The relative level of total proteins loaded in the lanes is illustrated by the intensity of tubulin. Histograms of ratios of the 7S and multimeric complexes to monomer in wild-type and *syx^3–69^* mutant flies are shown in (B). *, *p* < 0.05. (C and D) Western blots show the SNARE complexes, tubulin, syntaxin 1A, SNAP-25, and N-Syb from fly-head extract inputs, immunoprecipitates (pellets), and SDS head extracts. The head extracts used for immunoprecipitation were obtained from the wild-type (+/+) and the *syx^3–69^* mutant *(syx)* flies and incubated with Sepharose bead–coupled SNAP-25 antibodies. Inputs and precipitates were boiled in SDS sample buffer before loading. One of the SDS head-extract samples was not boiled to preserve the SDS-resistant SNARE complexes. The Western blot was sequentially probed with a syntaxin 1A antibody for SNARE complexes and syntaxin 1A monomers, a different SNAP-25 antibody for SNAP-25 (which also recognizes SNAP-24), an N-Syb antibody for N-Syb, and a tubulin antibody for tubulin. Note that tubulin is absent from pellet and that the SNARE complexes are only present in the unboiled SDS head extracts. SNAP-24 is present only in the input lanes and the SDS extracts; it is absent from the pellets because the IP antibody is specific for SNAP-25. Compared to the wild-type lanes, there are slightly more N-Syb, syntaxin 1A, and SNAP-25 in the precipitates from the *syx^3–69^* mutant *(syx)* mutant flies.

To test these structural predictions, we first investigated the biochemistry of SNARE complex assembly in the *syx^3–69^* mutant at room temperature. Unlike the results obtained at the restrictive temperature (Figure 4), our measurements showed that the average amount of the SDS-resistant 7S SNARE complex was significantly increased in the *syx^3–69^* mutant compared to that in the wild type *(CS)* at 22 °C (*n* = 50, *p* < 0.05) (Figure 6A and 6B). Similarly, the level of SNARE multimers was also significantly increased in the mutant (*n* = 9, *p* < 0.05). These results show that the level of SNARE complexes is increased in the *syx^3–69^* mutant. Concerned that the SDS-resistant SNARE complex is unique to neuronal SNAREs [37,38], we next used an alternative method to immunoprecipitate the SNARE complex from fly-head extracts using a polyclonal antibody against one of the SNARE components, SNAP-25 [39,40]. Our results showed that the SNAP-25 antibody readily and specifically precipitated syntaxin 1A and synaptobrevin, but not tubulin (Figure 6C). When normalized to the amount of proteins precipitated from the head extracts in the wild-type flies, the levels of syntaxin 1A, synaptobrevin, and SNAP-25 were all increased slightly (Figure 6D; *n* = 4). Even though these changes are not statistically significant, the trend is consistent with those observed for the SDS-resistant complexes.

### The T254I Mutation Stimulates the Rate of Constitutive Fusion at Synapses

**Figure 7:**
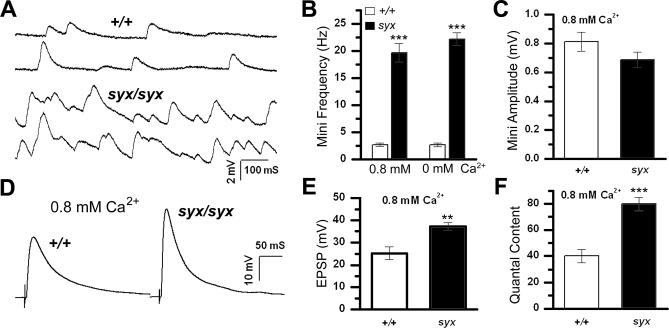
Both Constitutive Secretion and Ca^2+^-Triggered Vesicle Fusion Are Dramatically Enhanced in *syx^3–69^* Mutant Flies (A–C) The rate of spontaneous fusion of synaptic vesicles detected as mEPSPs (or minis) is significantly increased in *syx^3–69^* mutants compared to the wild-type control (+/+). Representative recordings of minis and histograms of mini frequency from the wild-type and the mutant larvae are shown in (A) and (B). The average frequency of minis is increased by 7-fold in the *syx^3–69^* mutant (B), whereas the average amplitude of these minis is similar (C). Note that the increase in the rate of constitutive secretion persists in saline containing 0 [Ca^2+^] (B). These and all other electrophysiological recordings were conducted at 19–20 °C. (D–F) The amplitude of EPSPs triggered by action potential–evoked Ca^2+^ entry is significantly increased in the *syx^3–69^* mutant. Representative traces of EPSPs, histograms of average EPSP amplitude, and quantal content from the wild type and the *syx^3–69^* mutant are shown in (D), (E), and (F), respectively. **, *p* < 0.01; ***, *p* < 0.001.

The level of the SDS-resistant SNARE complex has been shown to correlate well with the level of exocytosis [39,41,42]. We next tested whether this increase in the rate of SNARE complex assembly had any physiological effects on synaptic vesicle fusion. We recorded action potential–independent and constitutive (or spontaneous) miniature excitatory postsynaptic potentials (mEPSPs or minis) from third instar larval body-wall muscles innervated by motoneurons [43,44]. These mEPSPs are caused by constitutive secretion of glutamate from the nerve terminal [43]. Surprisingly, we found that the frequency of constitutive release was dramatically increased some 7-fold in the mutant (*n* = 9) compared to the wild type (*n* = 8; *p* < 0.001) (Figure 7A and 7B). The average mini amplitude was similar in both the *syx^3–69^* mutant (*n* = 11) and the wild-type larvae (*n* = 8; *p* > 0.1) (Figure 7C), suggesting that quanta and postsynaptic receptors likely remain normal. Immunocytochemical studies of glutamate receptors failed to show detectable differences between the mutant and the wild type (unpublished data). This mini recording was conducted in saline containing 0.8 mM Ca^2+^ and 1 μm TTX, which was also used for evoked synaptic potentials (below). The resting potential was not different between these two genotypes (−69.7 ± 1.2 mV, *n* = 8, for the wild type, and −69.4 ± 0.9 mV, *n* = 9, for the mutant; *p* > 0.5). In these and all other larval recordings shown in this study, the muscle input resistance (between 5–9 MΩ) did not differ between the wild-type and the mutant larvae.

To test whether this increase in mini frequency depends on extracellular Ca^2+^, we recorded minis in a Ca^2+^-free saline. The unusually high rate of spontaneous release remained in the *syx^3–69^* mutant in the absence of extracellular Ca^2+^ (*n* = 8), but significantly higher than that in the wild type (*n* = 8; *p* < 0.001) (Figure 7B). The resting potential was not different (−69.75 ± 1.18 mV, *n* = 8, for the wild type; −69.75 ± 0.85 mV, *n* = 8, for the mutant; *p* > 0.5). The lack of effects by Ca^2+^ removal on mini frequency is consistent with an earlier report showing that Ca^2+^-free saline plus EGTA did not alter mini frequency at the *Drosophila* larval NMJ [19]. Furthermore, mini frequency remained 13-fold higher in *syx^3–69^* mutants compared to the wild type in Ca^2+^-free saline containing the membrane-permeable Ca^2+^ chelator EGTA-AM (*n* = 4). These results indicate that the T254I mutant syntaxin 1A couples the formation of SNARE complexes with constitutive vesicle fusion even when the intracellular [Ca^2+^] is greatly reduced.

### The T254I Mutation Also Stimulates Ca^2+^-Evoked Vesicle Fusion, but Does Not Affect Vesicle Recycling

An increase in SNARE complex assembly could enhance Ca^2+^-evoked exocytosis. On the other hand, the dramatic increase in the rate of constitutive vesicle fusion could deplete the vesicle pool and reduce Ca^2+^-evoked release. To distinguish these possibilities, we recorded action potential–evoked excitatory postsynaptic potentials (EPSPs) from muscles bathed in 0.8 mM Ca^2+^ saline. We observed that the amplitude of evoked EPSPs was also significantly increased to 37 mV (*n* = 11) in the *syx^3–69^* mutant from 25 mV (*n* = 9) in the wild type (*p* < 0.005; Figure 7D and 7E). Because the average mini amplitude was not significantly different between the mutant and the wild type (*p* > 0.1), this increase in EPSP amplitude most likely reflected an enhancement in presynaptic release. Factoring in the respective average mini amplitude in these flies and after correction of EPSP amplitudes for nonlinear summation [45,46], there was a 2-fold increase in quantal content from 40.0 (*n* = 8) in the wild type to 79.9 (*n* = 11) in the mutant (Figure 7F; *p* < 0.001). As with the mini measurement, there was no difference in resting potentials of the muscle fiber between the wild type and the mutant. These results indicate that the average number of synaptic vesicles undergoing exocytosis induced by an action potential is significantly increased in the *syx^3–69^* mutant.

**Figure 8:**
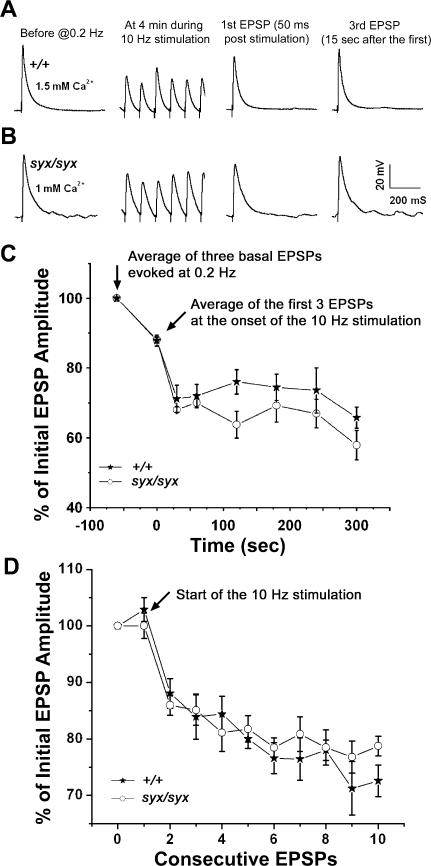
The T254I Mutation in the Syntaxin 1A Does Not Affect Synaptic Vesicle Recycling (A and B) Representative traces of EPSPs from the wild-type (+/+ [A]) and the *syx^3–69^* mutant larvae (*syx/syx* [B]) before, 4 min during, and 50 ms after a 5-min repetitive stimulation at 10 Hz. The nerve is stimulated at 0.2 Hz prior to and after the repetitive stimulation. Note that the extracellular [Ca^2+^] was adjusted to 1.5 mM in the wild type and 1 mM in the mutant so that their basal transmitter release is similar. (C) EPSP amplitudes decline after the onset of 10-Hz repetitive stimulation over a 5-min period. The average amplitude of three consecutive EPSPs is obtained at the onset of the 10-Hz stimulation (time zero), at 30 sec, and at every minute thereafter. These average values are then normalized to the basal EPSP amplitude evoked at 0.2 Hz prior to the repetitive stimulation. The steady-state level is reached when the rate of exocytosis equals that of vesicle recycling. The decline rate is essentially similar between the two genotypes, suggesting that the *syx^3–69^* mutant does not differ significantly from the wild type in vesicle recycling. (D) The RRP of synaptic vesicles can be depleted within a few stimuli. This plot shows the relatively decline of the EPSP amplitude following the first ten stimuli at 10 Hz. The nearly identical decline rate suggests that vesicle docking and priming is not reduced in the *syx^3–69^* mutant despite the extraordinarily high rate of spontaneous fusion.

Another possibility predicted by our structural modeling is that the T254I mutation may slow vesicle recycling by stabilizing post-fusion *cis* SNARE complexes. To test this hypothesis, we repetitively stimulated the motor nerve at 10 Hz for a prolonged period (5 min). We adjusted the extracellular [Ca^2+^] such that the initial EPSP amplitude was similar between the wild-type control (at 1.5 mM Ca^2+^) and the *syx^3–69^* mutant (at 1 mM Ca^2+^) (Figure 8A and 8B). At these [Ca^2+^], the resting potential was −76.5 ± 2.6 mV (*n* = 4) and −75.6 ± 1.6 mV (*n* = 6) for the wild type and the mutant, respectively. The basal release was 52.8 ± 1.0 mV (*n* = 4) and 49.5 ± 1.6 mV (*n* = 6) for the wild type and the *syx^3–69^* mutant (*p* > 0.1), respectively. As previously shown, there was an initial, rapid decline in the amplitude of EPSPs after the onset of the moderate stimulation at 10 Hz [47]. The EPSP amplitude then reached a steady-state level approximately 60%–65% of single-pulse–induced EPSPs (Figure 8C). Under such stimulation conditions, the steady-state release level is thought to reflect the balance between vesicle recycling and exocytosis [47]. There were no statistical differences in the rate of EPSP decline or the steady-state levels between the wild-type and the *syx^3–69^* mutant larvae (*p* > 0.05). EPSPs recovered at a similar rate after the 5-min stimulation (Figure 8C). These results suggest that the T254I mutation does not have a detectable effect on synaptic vesicle recycling.

At *Drosophila* NMJs, the readily releasable pool (RRP) of synaptic vesicles is estimated to be 230, which can be rapidly depleted within a few stimuli [47]. We examined the RRP by measuring the relative amplitude of the first ten EPSPs after the onset of the 10-Hz stimulation. Our results showed no significant difference between the wild-type (*n* = 4) and the *syx^3–69^* mutant flies (*n* = 5; Figure 8D). Thus, the RRP of synaptic vesicles is not reduced in the *syx^3–69^* mutant despite the extraordinarily high rate of spontaneous fusion rate. Taking into consideration the high rate of spontaneous vesicle fusion, it is reasonable to assume that vesicle docking is, in effect, increased in *syx^3–69^* mutants.

### The T254I Mutation Increases Evoked Transmitter Release in a Dominant Fashion and across a Wide Spectrum of [Ca^2+^]

**Figure 9:**
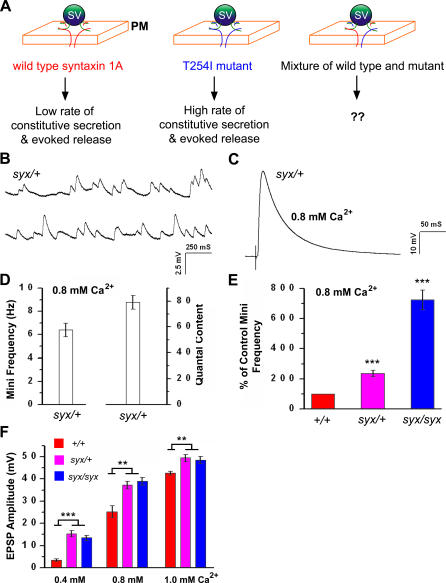
The T254I Mutation Exerts Dominant Positive Effects on Both Constitutive and Ca^2+^-Triggered Vesicle Fusion in *syx^3–69^* Heterozygotes (A) Models of multimeric SNARE complexes found in the wild type (+/+), the homozygous *syx^3–69^* mutant *(syx/syx),* and the heterozygous *syx^3–69^* mutant (*syx/*+). Oligomerization of a mixture of the wild-type and the mutant SNARE complex predicts that the T254I mutant syntaxin 1A has dominant positive effects on vesicle fusion. The wild-type syntaxin 1A is illustrated in red, whereas the T254I mutant syntaxin 1A is in blue. For simplicity, SNAP-25 is omitted from these models. PM, plasma membrane; SV, synaptic vesicle. (B–D) Representative traces of minis and evoked EPSPs in the heterozygous larvae are shown in (B) and (C), respectively. The average mini frequency and quantal content are shown in (D). (E) The histogram shows that the normalized mini frequency in the heterozygote is significantly higher than that in the wild type, but much lower than that in the homozygous mutant. ***, *p* < 0.001. (F) A histogram of the average EPSP amplitude recorded from the wild type (+/+), the heterozygote (*syx/*+), and the homozygote *(syx/syx)* at three different Ca^2+^ concentrations: 0.4 mM, 0.8 mM, and 1 mM. At these Ca^2+^ concentrations, the amplitude of EPSPs in the wild type is consistently lower than those in the heterozygote and the homozygote. Note that the difference between the wild type and mutants (the heterozygote and the homozygote) appears more dramatic at lower Ca^2+^ concentrations. At higher Ca^2+^ concentrations, this difference becomes smaller because EPSPs reach the “ceiling” set by the reversal potential. The amplitude of EPSPs is similar between the heterozygote and the homozygote. **, *p* < 0.01; ***, *p* < 0.001.

Oligomerization of the 7S SNARE complex into high molecular weight complexes is proposed to be essential for vesicle fusion [39]. This implies that multiple SNARE complexes are required to promote vesicle fusion. The precise number of SNARE complexes required for vesicle fusion is unknown, but is estimated to be between three and 15 pairs [3,48]. Hence, one could envision a scenario in which the I254 mutant syntaxin 1A exerts a dominant positive effect on synaptic vesicle fusion in heterozygous mutant flies (i.e., flies that also have one copy of the wild-type syntaxin 1A) by acting as part of the multimeric SNARE complex (Figure 9A). To test this hypothesis, we generated heterozygous *syx^3–69^/*+ larvae. The resting potential of the muscle fiber in *syx^3–69^/*+ larvae was −70.9 mV (*n* = 7) at 0.8 mM Ca^2+^, which is not significantly different from those in the wild type (+/+; −69.8 mV) and the *syx^3–69^/syx^3–69^* homozygous mutant (−69.4 mV) under the same [Ca^2+^] (*p* > 0.3). The frequency of spontaneous fusion (6.4 Hz; *n* = 7) was significantly higher than that in the wild type (2.65 Hz, *p* < 0.001), but much lower than that in the homozygote (19.67 Hz, *p* < 0.001). This observation is consistent with the working model that I254-containing syntaxin 1A has a dominant positive effect on vesicle fusion.

We next recorded evoked release in heterozygotes and showed that the amplitude of evoked EPSPs (39 mV; *n* = 9) was similar to that in the homozygote (37.3 mV; *p* > 1), but significantly higher than that in the wild type (25.3 mV, *p* < 0.001) (Figure 9B–9E). These results indicate that the T254I mutant syntaxin 1A also has a dominant positive effect on Ca^2+^-triggered vesicle fusion. However, it is clear that dilution of the mutant SNARE complex by the wild-type syntaxin 1A does not reduce evoked release, as it does to constitutive secretion. This observation is inconsistent with the possibility that the T254I mutant syntaxin 1A enhances Ca^2+^ influx, as one would expect a greater increase in evoked release in the homozygote. A likely explanation we suggest is that the T254I mutant syntaxin 1A stimulates the formation of SNARE complexes in a dominant fashion. For a given level of SNARE complexes, the energy barrier for fusion correlates negatively with the amount of the T254I mutant syntaxin 1A. Although this energy barrier is increased for constitutive fusion in the heterozygote compared to the homozygote, this barrier should be overcome easily by the rise of intraterminal Ca^2+^. Our measurement of the SDS-resistant complex confirmed that the amount of SNARE complex was similar between homozygotes and heterozygotes (Figure S4).

This model further predicts that the increase in evoked release should occur in both homozygotes and heterozygotes at both low and high [Ca^2+^]. To this end, we recorded EPSPs at two additional Ca^2+^ concentrations (1 mM and 0.4 mM). These Ca^2+^ concentrations did not alter resting potentials (unpublished data), but they did affect transmitter release (Figure 9F). At 1 mM Ca^2+^, the average EPSP amplitude was similar in the heterozygote (*syx/*+*,* 48.6 mV, *n* = 7) and homozygote (*syx/syx,* 49.5 mV, *n* = 6), but was consistently larger than the wild type (+*/*+, 42.7 mV, *n* = 8; *p* < 0.01). At 0.4 mM Ca^2+^, the amplitude of EPSPs in the wild type was quite small (3.5 mV, *n* = 9). In comparison, the amplitude of EPSPs was significantly larger in both heterozygotes (13.5 mV, *n* = 8) and homozygotes (15.4 mV, *n* = 9) of *syx^3–69^* (*p* < 0.001). The average amplitude of EPSPs was then compared with those seen at 0.8 mM [Ca^2+^] (Figure 9F). The relatively smaller increase of EPSP amplitude at increasingly higher [Ca^2+^] reflects the ceiling effect due to non-linear summation. Nonetheless, these results show that evoked release is dramatically enhanced in I254-containing flies at a wide spectrum of extracellular [Ca^2+^].

### The Effect of the Mutant Syntaxin 1A Is Specifically Rescued by Neuronal Expression of the Wild-Type Syntaxin 1A

**Figure 10:**
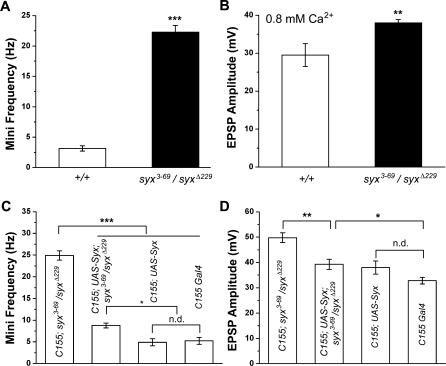
Genetic and Electrophysiological Evidence That Neuronal Overexpression of the Wild-Type Syntaxin 1A Specifically Rescues the *syx^3–69^* Mutant Phenotype (A and B) The increase in mini frequency and evoked EPSP amplitude persists in larvae carrying only one copy of the *syx^3–69^* mutant gene. This mutant fly *(syx^3–69^*/*syx^Δ229^)* is generated by placing one mutant gene in *trans* to the null mutation *(syx^Δ229^)* in the *syntaxin* locus. These electrophysiological defects are nearly identical to those found in the *syx^3–69^*/*syx^3–69^* homozygote, but different in mini frequencies from the *syx^3–69^*/+ heterozygote. These results demonstrate that the mutant phenotype is specifically caused by the *syx^3–69^* mutation. **, *p* < 0.01; ***, *p* < 0.001. (C and D) Neuronal overexpression rescues the physiological defects observed in *syx^3–69^* mutants. In C155 Gal4 background, the *syx^3–69^*/*syx^Δ229^* mutant only expresses one copy of the T254I mutant protein. Under such circumstances, the C155 Gal4; *syx^3–69^*/*syx^Δ229^* larvae display an extraordinarily high frequency of minis and enhanced amplitude of EPSPs. Neuronal overexpression of the wild-type syntaxin 1A (UAS-Syx driven by C155 Gal4) in the *syx^3–69^*/*syx^Δ229^* mutant background dramatically reduces mini frequency to a level slightly higher than that in the wild type (i.e., C155 Gal4; UAS-Syx or C155 Gal4 flies) (C). The EPSP amplitude is also similarly reduced to the wild-type level (D). Importantly, overexpression of the wild-type syntaxin 1A in the wild-type background (i.e., C155 Gal4; UAS-Syx) has little effect on both the mini frequency and evoked EPSP amplitude compared to the C155 Gal4 flies. Thus, the rescuing effect on vesicle fusion by the wild-type syntaxin 1A is specific to the T254I mutant syntaxin 1A in the *syx^3–69^* mutant. *, *p* < 0.05; **, *p* < 0.01; ***, *p* < 0.001.

The possibility remains that the dominant positive effects we have seen in the heterozygote could result from a second site mutation elsewhere rather than the T254I mutation in the *syntaxin* locus. To address this concern, we generated transheterozygous flies (*syx^3–69^*/*syx^Δ229^*) in which the *syx^3–69^* mutant chromosome was placed in *trans* to a null *syntaxin* mutation *(syx^Δ229^)* [23]. In *syx^3–69^*/*syx^Δ229^* mutants, the mini frequency was 22.3 Hz (*n* = 9), which is significantly higher than that in the wild-type larvae (3.2 Hz, *n* = 8; *p* < 0.0001) (Figure 10A). At 0.8 mM Ca^2+^, the evoked EPSP amplitude was also significantly increased to 37.9 mV (*n* = 9) from 29.5 mV (*n* = 8) in the wild-type larvae (*p* < 0.01) (Figure 10B). The resting potential of the mutant animal (−72.4 mV) was similar to that (−73.8 mV) in the wild-type larvae. These results are highly similar to those found in the *syx^3–69^*/*syx^3–69^* homozygote. Along with the molecular evidence presented earlier, these results provide further genetic and electrophysiological evidence that the effects we have observed in the *syx^3–69^* mutant is specifically caused by the T254I mutation in the *syntaxin* gene.

To further demonstrate indeed the “neutralizing” effect on the T254I mutant syntaxin 1A is mediated by the wild-type syntaxin 1A in the heterozygote, we then performed a genetic rescue experiment in which we selectively expressed the wild-type syntaxin 1A gene in postmitotic neurons using the Gal4-UAS binary system [49,50]. When the wild-type syntaxin 1A gene was expressed in the wild-type background (C155 Gal4/+; UAS-Syx 1A/+), it had no significant effect on either the constitutive secretion rate or the amplitude of evoked EPSPs compared to those in the wild-type larvae carrying the pan neuronal Gal4 driver (C155 Gal4, *n* = 7; *p* > 0.05) (Figure 10C and 10D). However, neuronal overexpression of the wild-type syntaxin 1A in the *syx^3–69^*/null mutant background (i.e., C155 Gal4/+; UAS-Syx 1A/+; *syx^3–69^*/*syx^Δ229^* [23]) resulted in a dramatic reduction in the frequency of constitutive secretion to 8.8 Hz (*n* = 14) from 24.9 Hz (*n* = 10) in C155 Gal4/+; *syx^3–69^*/*syx^Δ229^* mutant larvae (*p* < 0.001). This rate is significantly higher than that in the C155 Gal4 larvae (5.3 Hz, *n* = 7) or overexpression alone (C155 Gal4/+; UAS-Syx 1A/+, 4.9 Hz, *n* = 9) (*p* < 0.05). Overexpression of the wild-type syntaxin 1A in the *syx^3–69^*/null mutant background also significantly reduced the average amplitude of EPSPs from 49.8 mV (*n* = 10) in C155 Gal4/+; *syx^3–69^*/*syx^Δ229^* mutant larvae to 39.3 mV (*n* = 14). This EPSP amplitude is similar to that in the C155 Gal4/+; UAS-Syx 1A/+ background (38.0 mV, *n* = 9), but significantly higher compared to that in the C155 Gal4 larvae (32.8 mV, *n* = 7). These results demonstrate that neuronal expression of the wild-type syntaxin 1A rescues the mutant phenotype by specifically neutralizing the dominant positive effects on both constitutive and evoked secretion induced by the T254I mutant syntaxin 1A.

## Discussion

This study reports the behavioral, electrophysiological, biochemical, genetic, structural, and molecular results from a re-investigation of the *syx^3–69^* mutant in *Drosophila*. These findings contradict an earlier report [21] on both the experimental evidence and conclusions concerning the effects of the T254I mutation in syntaxin 1A on synaptic transmission. Multiple lines of evidence demonstrate that the T254I mutation in the *syx^3–69^* mutant fly blocks neither synaptic transmission nor SNARE complex assembly at restrictive temperatures. More importantly, we have gone steps further by revealing an evolutionarily conserved structural feature among syntaxin orthologs in regulating both constitutive secretion and Ca^2+^-regulated exocytosis.

### Evidence That the T254I Mutation Enhances Both Constitutive and Evoked Secretion

One of the major new findings from this study is that the T254I mutant syntaxin 1A in the *syx^3–69^* mutant dramatically stimulates vesicle fusion. At the restrictive temperature, the *syx^3–69^* flies exhibit uncontrolled hyperactivities and enhanced neuronal firing. At the permissive temperature, SNARE complex assembly is moderately enhanced, whereas the rate of constitutive vesicle fusion is dramatically increased in the *syx^3–69^* mutant. Importantly, this enhancement of constitutive secretion persists in Ca^2+^-free saline and when intracellular Ca^2+^ is further reduced by chelation. This implies that spontaneous vesicle fusion is less dependent upon Ca^2+^, a conclusion consistent with those reported in a number of synapses [16–18], including the *Drosophila* NMJ [19]. Although we do not suggest that vesicle fusion is absolutely independent of intracellular Ca^2+^, our studies support the notion that the T254I mutation makes vesicle fusion more efficient, regardless of whether it is constitutive or Ca^2+^-regulated fusion.

Another major finding is that despite the high rate of constitutive fusion, the vesicle pool is not depleted, implying that vesicle docking or priming is enhanced in the *syx^3–69^* mutant via a yet unidentified mechanism. Consistent with the increase in mini frequency, evoked transmitter release is significantly increased in the *syx^3–69^* mutant. Thus, the T254I mutation stimulates both constitutive and evoked vesicle fusion. This increase in evoked transmitter release correlates well with the enhanced assembly of SNARE complexes in the mutant fly.

The third interesting finding is that the T254I mutant syntaxin 1A exerts a dominant positive effect on vesicle fusion. In heterozygous *syx^3–69^* mutant (*syx^3–69^*/+), the rate of spontaneous fusion is slightly higher than that in the wild-type larvae, whereas evoked release remains at the homozygote level. Two lines of genetic and electrophysiological evidence suggest that this dominant positive effect is specifically associated with the T254I mutation in the *syntaxin* locus. First, the dominant positive effect persists in larvae carrying only one copy of the T254I mutation in the null mutant background (i.e., *syx^3–69^*/*syx^Δ229^*). Second, neuronal overexpression of the wild-type syntaxin 1A effectively rescues the effect of the T254I mutant in the *syx^3–69^*/null mutant background. It is important to note that neuronal overexpression of the wild-type syntaxin 1A protein in the wild-type background has little effect on both spontaneous and evoked vesicle fusion. Therefore, the counterbalance exerted by the wild-type syntaxin 1A is specific to the T254I mutant syntaxin 1A. Taken together, these observations lend further support to the notion that the I254 mutant syntaxin 1A is more efficacious than the wild-type T254 syntaxin 1A in promoting vesicle fusion.

### Potential Mechanisms Underlying the Effect of the T254I Mutant Syntaxin 1A

How might the T254I mutation exert such a dramatic effect on vesicle fusion? The precise mechanism is unknown; however, we believe the effect of the mutant protein can be better explained by examining the structural impact of the point mutation on the SNARE complex. The formation of the SNARE complex is generally accepted as an essential step in vesicle fusion. This conclusion is supported by considerable evidence accumulated over the last decade using a variety of experimental methods, including the use of specific neurotoxins to cleave SNARE proteins, and genetic mutations or deletion of SNARE genes [1–3]. Based on structural and functional studies of the core complex [6–8], it has been postulated that the assembly of the SNARE complex involves a “zippering” process in which complex formation starts at the N-termini of the four helices, followed by zippering of the core “layer” of the SNARE bundle towards the C-terminal bundles. The process of zippering is also believed to provide the energy necessary to bring the vesicle close to the plasma membrane [2,3,9]. To date, most of the data supporting this zipper model came from observations of “loose” and “tight” states of SNARE complexes in neuroendocrine cells [51], at crayfish neuromuscular synapses [52], and in liposome fusion [53]. It is also indirectly supported by genetic mutations of the helical region of SNAREs (see discussions in [7]) and by the ability of inhibitory peptides of the helical region of SNAREs to block both core complex assembly in vitro and transmitter secretion in PC12 cells [54,55]. At present, both the precise mode of SNARE complex formation [56] and the role of the complex in vesicle fusion [3,57] are not fully resolved. Nonetheless, the zipper model serves as a good starting point for experimental testing of SNARE structure and function.

The T254I mutation is located at a strategic location near the end of the zipper, a presumed final step before vesicle fusion takes place. We have made three interesting observations of the +7 layer by sequence and structural comparison. First, with a few exceptions, nearly all syntaxins involved predominantly in regulated vesicle exocytosis at synapses or neurosecretory cells have a common hydrophilic residue, threonine, at position 254 in the +7 layer. In contrast, most syntaxins acting in the constitutive secretory pathways have one of the highly conserved hydrophobic residues (I, L, or V). Second, there is a conserved switch in the +7 layer packing among SNARE complexes used in different secretory pathways. This layer is loosely packed in “synaptic” SNAREs, but tightly bundled together in “constitutive” SNAREs, where hydrophobic residues (I, L, or V) may enhance direct intermolecular interactions among the four α-helices. Third, our structural modeling suggests that the T254I mutant +7 layer is more tightly packed than is the wild type, and that it resembles more the tight packing found in the endosomal SNARE core complex [20].

Based on our sequence and structural analyses, we favor the idea that a structural alteration of the +7 layer induced by the T254I mutation in syntaxin 1A may best account for our experimental observations. The extraordinarily high rate of spontaneous fusion detected in the *syx^3–69^* mutant appears to support the “zipper model” or a modified zipper model [56], suggesting that tightening the SNARE complex does promote vesicle fusion. That overexpression of T254 syntaxin 1A specifically counteracts I254 mutant syntaxin 1A in vesicle fusion implies that the relatively loose packing of the +7 layer containing the wild-type syntaxin 1A may serve as an internal brake to dampen vesicle fusion. Once this brake is removed by the T254I substitution, the mutant SNARE complex lowers the energy barrier for vesicle fusion beyond a point of no return in a manner that is relatively less dependent on intracellular Ca^2+^ [15–19]. This working model also explains why evoked release is enhanced in both homozygotes and heterozygotes.

We should stress that our results do not permit us to conclude whether or not SNARE complexes directly mediate vesicle fusion. Although pairing of SNARE proteins has been shown to mediate liposomal vesicle fusion in vitro [57], the rate of liposomal fusion is slow. More importantly, new evidence suggests that SNARE proteins alone may bring the membranes in close apposition, but do not drive vesicle fusion under more physiological conditions [58]. The failure to mediate fusion in vitro suggests that other factors may either assist SNARE function or directly mediate vesicle fusion in vivo. Consistent with this idea, the vesicular ATPase (Vo) and synaptotagmin I have been reported to act either downstream of, or synergistically with, the SNAREs in vesicle fusion [50,59]. It is interesting to note that a G50E mutation in the N-terminal domain of SNAP-25 has previously been found to enhance both constitutive secretion and Ca^2+^-evoked release in *Drosophila* at the permissive temperature [60]. Unlike the T254I mutation, this G50E (G43E in mammals) mutation is thought to cause a conformation change of SNAP-25 such that the mutant SNARE complex is more ready to mediate vesicle fusion. The precise mechanism by which the G50E mutation promotes vesicle fusion remains to be resolved. Nonetheless, the T254I and G50E mutations offer two alternative structural changes to promote SNARE-mediated vesicle fusion. It is evident that much is still to be discovered about SNARE structure and function. The results presented here reveal a novel intrinsic mechanism by which SNARE-mediated vesicle fusion is regulated. These findings not only advance the understanding of synaptic transmission, but also have broad implications on vesicle fusion at different cellular pathways.

## Materials and Methods

### Fly strains.

The *syx^3–69^* mutant fly [21] was obtained from the laboratories of Drs. Troy Littleton (Massachusetts Institute of Technology), Barry Ganetzky (University of Wisconsin-Madison), and Leo Pallanck (University of Washington). This mutant line was maintained on a balancer chromosome (TM6B) and out-crossed to prevent potential accumulation of modifiers. The *syntaxin* null allele (*syx^Δ229^* [23]) and UAS-Syntaxin [50] flies were obtained from Dr. Hugo Bellen's laboratory. The wild-type *Canton S* (*CS* or +/+) strain, *Shibire^ts1^ (Shi^ts1^),* and *paralytic^ts1^ (para^ts1^)* originally obtained from the Bloomington *Drosophila* Stock Center were maintained in B. Z.'s lab. Flies were cultured on standard fly medium at room temperatures (∼20–22 °C). Unless otherwise specified, 3- to 5-d-old flies of both sexes were used in the adult experiments described.

### Structural modeling and molecular dynamics simulations.

The modeling of the T251I mutation (equivalent to the *Drosophila* T254I mutation) in syntaxin 1A was accomplished using PyMol [61]. The appropriate residue was modified (mutated) in the SNARE complex (1SFC, chain B). Ray tracing for Figure 5C was also performed with PyMol.

All dynamics calculations were carried out with GROMACS v3.3, in which the Gromacs 96 force field was used throughout [35,36]. The X-ray structure of the SNARE complex (chains A[synaptobrevin], B[syntaxin], C[SNAP-25], and D[SNAP-25] of 1SFC) was used as the starting model. Ordered water molecules and ordered divalent ions were excluded from this calculation. Hydrogen atom positions were calculated with the pdb2gmx program provided by GROMACS, resulting in 3,001 atoms (excluding SPC water molecules) in the native structure. Sixteen sodium ions were selected by the genion program included with GROMACS to negate the net negative charge of the SNARE bundle. A rectangular box of water (the SPC water model) extending 20 Å in every direction from the boundary of the protein component was calculated by the editconf program included with GROMACS [35,36]. Initially, the protein structure was minimized until convergence. Position-restrained molecular dynamics was used to equilibrate (simulation time of 20 ps) the water molecules with the protein. After energy minimization of the entire system was completed (protein + solvent + counter-ions), 5 ns of molecular dynamics trajectories were computed at 300 K. Once the system was equilibrated, a representative model was extracted from the trajectory (at 2 ns) and examined (Figure 5).

### Preparation and detection of the SDS-resistant SNARE complex.

The methods described by Tolar and Pallanck (1998) [11] and by Littleton et al. (1998) [21] were closely followed for fly treatments and for the extraction of SDS-resistant SNARE complexes. Briefly, adult *syx^3–69^* and *CS* flies were exposed to 38 °C for 20 min or kept at 22 °C, and then rapidly frozen in liquid nitrogen. Heads were separated from the body by brief vortexing and approximately 20 heads were collected on a sheet of paper under a constant superfusion with liquid nitrogen. Taking care to avoid introduction of air bubbles, fly heads were ground gently in 100-μl SDS sample buffer with a plastic pestle in an Eppendorf tube followed by centrifugation. The supernatant was collected, diluted to a final concentration at 0.25–0.5 heads/10 μl in sample buffer, and loaded onto gels at 10–15 μl per well. Samples were run on a discontinuous SDS-polyacrylamide gel: a 4% stacking gel, an upper 7.5%–8% resolving gel, and a lower 18% resolving gel to minimize excessive transfer of the monomer [11]. Commercial 4%–18% gradient gels were also used in one fourth of the experiments. Proteins were transferred to nitrocellulose membranes by running at 30 V overnight in a 4 °C room, as outlined by the manufacturer's instructions (Bio-Rad, Hercules, California, United States). Membranes were probed with a monoclonal antibody to syntaxin 1A (8C3; 1:100) [21]. Bands representing monomeric syntaxin 1A and the 7S complex were detected by enhanced chemiluminescence (ECL; GE Healthcare, Amersham Biosciences, Piscataway, New Jersey, United States) and quantified with ImageJ (National Institutes of Health [NIH], http://rsb.info.nih.gov/ij/). The SNARE complex level is expressed as a 7S complex to monomer (syntaxin) ratio and normalized to that in control *(CS)* flies at room temperature. To obtain oligomeric or multimeric complexes of the 7S complex [39], we either prolonged the exposure time of the film or loaded up to one head equivalent volume onto the gel. The ratio of the multimeric complex to the syntaxin 1A monomer was determined similarly to that for the 7S complex [11]. In most experiments, the blot was re-probed for tubulin to ensure that the protein loading level was similar in each lane.

### Immunoprecipitation of SNARE complexes.

Twenty**-**one adult fly heads were collected from wild-type *(CS)* and *syx^3–69^* mutant flies (4- to 5-d-old) and ground on ice in 100-μl immunoprecipitation (IP) buffer containing 150 mM NaCl and 20 mM Tris (pH 7.5), and mini complete protease inhibitors (which were added at one tablet/10-ml IP buffer; Roche, Basel, Switzerland). The fly-head extract was mixed well with 4-μl 25% Triton X-100 (BioRad, Hercules, California, United States) and incubated for 15 min on ice. After a brief and gentle spin to remove cuticle debris, 20 μl of the supernatant was saved as “input” for control loading. The remaining supernatant was incubated with 10-μl sera against the *Drosophila* SNAP-25 (rabbit, N-terminal [40], and mixed with 20-μl prewashed CL-4B protein A Sepharose beads (GE Healthcare, Amersham Biosciences) by gentle rotation for 1–3 h at 4 °C. After removing the supernatant, the beads were washed four times with the IP buffer. The immunoprecipitates were eluted by boiling the beads in 50-μl sample buffer. The precipitates along with “input” were resolved on standard SDS gel and subject to Western blot analysis. SDS-resistant complexes were prepared separately and included on the gel as additional controls. The blot was sequentially probed with the 8C3 syntaxin 1A monoclonal antibody, a neuronal synaptobrevin (N-Syb) polyclonal antibody (guinea pig [62]), and a different SNAP-25 antibody (which also recognizes the close homolog SNAP-24 [40]). To ensure the specificity of immunoprecipitation, the blot was also probed with an antibody to α-tubulin (Sigma, St. Louis, Missouri, United States). The ECL method was used for protein detection. The band intensity was quantified with ImageJ (NIH).

### Electrophysiology.

The standard method of third instar larval electrophysiology described by Jan and Jan (1976) [43] and by Stewart et al. (1994) [63] was used to record spontaneous mEPSPs and action potential–evoked EPSPs in current clamp mode. The saline [Ca^2+^] was 0 or 0.8 mM for mEPSP recordings and 0.4, 0.8, 1, or 1.5 mM for EPSP recordings (specified in the text). A total of 1 μM tetrodotoxin was added to the saline to block action potentials when minis were recorded [44,64]. Because of the unusually high rate of spontaneous minis in the *syx^3–69^* mutant larvae, many minis were clustered together or on top of one another. This made it difficult to ascertain the amplitude of individual minis. In this case, minis were analyzed following an EPSP after the membrane potential had returned to pre-stimulation levels. Quantal content was determined as the ratio of the average EPSP amplitude and the average mini amplitude after correction of EPSP amplitude for nonlinear summation following the methods described by Stevens (1976) [45] and Feeney et al. (1998) [46]. Corrected EPSP amplitude = E{Ln[E/(E − recorded EPSP)]}, where E = difference between reversal potential and resting potential. The reversal potential used in this correction was 0 mV [46]. For simplicity, the average amplitude of EPSPs presented in Figures 9F, 10B, and 10D was not corrected for nonlinear summation. The room temperature was 19–20 °C.

Mini recordings were also performed in larvae treated with EGTA-AM (Invitrogen, Molecular Probes, Carlsbad, California, United States). The final concentration of EGTA-AM was 10 μm in Ca^2+^-free HL-3 saline, diluted from a 40 mM stock in 20% Fluronic F-127 (a low-toxicity dispersing agent) in dimethyl sulfoxide (DMSO). The preparation was incubated in the EGTA-AM saline at room temperature for 30 min and washed with Ca^2+^-free saline prior to recording. The effect of Ca^2+^ chelation was monitored at the end of the mini recording. Upon switching back to 0.8 mM Ca^2+^ saline, evoked release was dramatically reduced (unpublished data), suggesting most, if not all, of the large number of Ca^2+^ ions evoked by an action potential were chelated by the intraterminal EGTA. Control experiments with Ca^2+^-free saline containing the same final concentration (0.025%) of the Fluronic F-127/DMSO solvent were also conducted.

For the vesicle depletion assay, we used relatively higher concentrations of Ca^2+^ (1.5 mM for the wild type and 1 mM for the *syx^3–69^* mutant) to ensure achieving a rapid decline of EPSP amplitudes. Basal release was monitored at 0.2 Hz prior to a 5-min repetitive stimulation at 10 Hz. Usually, the recovery from the 10-Hz stimulation was also monitored by 0.2-Hz stimulation immediately afterwards. The basal amplitude of EPSPs was averaged from at least three consecutive EPSPs and used to normalize the average EPSP amplitude during the 10-Hz stimulation. At the onset of repetitive stimulation, three consecutive EPSPs were used to give the average amplitude at time 0, at 30 sec, and at every minute during the remaining the 10-Hz stimulation periods. The normalized plot, as shown in Figure 8C, allows one to estimate the decline rate and vesicle pools. The first ten EPSPs were also analyzed to estimate the depletion rate of the RRP.

The method of Tanouye and Wyman (1980) [31] was adapted for stimulating giant fiber neurons and for recording synaptic potentials and action potentials in DLMs. Flies were mounted on a slide with dental wax. Sharp glass microelectrodes (25 MΩ, filled with 3 M KCl) were used to record intracellularly from DLMs, whereas the giant fiber neurons were stimulated with a sharp tungsten electrode placed either inside the compound eye or in the cervical connective (1–6 V, 120-μs duration). A homemade temperature stage was used to rapidly (within 45 s) increase the recording chamber to 38 °C. The temperature probe was placed in dental wax next to the mounted fly to ensure the accuracy of the set-point temperature. The fly chamber was then rapidly cooled to room temperature by pumping ice-cold water through the metal stage. Wild type *(CS), syx^3–69^,* and *Shi^ts1^* were used for this set of experiments. The room temperature was 19–20 °C.

For ERG recordings, 2- to 3-d-old flies *(CS, Shi^ts1^,* or *syx^3–69^)* were mounted on a slide with modeling clay and placed on a temperature-controlled stage. A sharp tungsten electrode was inserted gently in the thorax or abdomen of the fly and served as a reference electrode. A sharp glass microelectrode was inserted just through the cuticle into the compound eye. The fly was then allowed to adapt to the dark for a few minutes. ERGs were evoked by rapidly exposing the eye to white light for a brief duration (1–2 s). The fly chamber temperature was raised to 38 °C using a homemade temperature controller. To ensure the accuracy of the temperature experienced by the fly, the monitor probe was placed as close as possible to the experimental fly. Another experimental fly was mounted beside the fly being recorded so that it could be monitored for paralysis during 38 °C and recovery after the temperature was returned to the permissive temperature.

### Kinetics of recovery from paralysis.

Flies were incubated at 38 °C in water-warmed glass vials for 10 min and then transferred to a sheet of paper placed on the lab bench for recovery at room temperature (∼22 °C). The time and number of flies capable of standing were recorded and plotted. A total of 230 *syx^3–69^* flies were tested in 17 different trials (10–15 flies per trial). Wild-type *(CS)* flies were used as controls (+/+) in a few trials, and they were not paralyzed at this temperature.

### Videos of *syx^3–69^* and *Shi^ts1^* in response to giant fiber stimulation.

Flies (*syx^3–69^* and *Shi^ts1^,* 1- to 2-d-old) were mounted with their ventral sides up on a slide with modeling clay and viewed with a dissection microscope at 100×. Flies were placed in a chamber whose temperature was rapidly raised to 38 °C within 45 s by a homemade temperature controller and rapidly cooled to 20 °C (within 1 min) by circulating ice-cold water around the chamber. A pair of sharp tungsten electrodes was placed into the compound eyes to electrically stimulate the giant fiber neurons in the brain (1–6 V, 100-μs duration, 5 Hz). Spontaneous and evoked leg movements of these flies were recorded using a digital camera. Videos S4 and S5 are on *syx^3–69^* and *Shi^ts1^,* respectively. Still clips from these videos are presented in Figure 2C.

### Statistics.

Results are presented as mean ± standard error of the mean (SEM). The paired Student *t*-test was used to analyze the level of SNARE complexes, whereas the unpaired *t*-test was used to treat the electrophysiological results. In all cases, differences of *p* < 0.05 were considered statistically significant.

## Supporting Information

Figure S1 Alignments of Amino Acids in the 0 to +8 Layers of the SNARE Core Complex in Syntaxin Orthologs in Yeast and *Arabidopsis*
Top panel: Examples of syntaxin orthologs from the different cellular compartments in the yeast are shown here. Note that, with the exception of SSO1 and SSO2, all syntaxins functioning in intracellular compartments have a conserved leucine at a position equivalent to 254 in *Drosophila* syntaxin 1A.
Bottom panel: *Arabidopsis* has a large number of syntaxin orthologs. With the exception of syntaxin 61 (which has a valine at position 254), all others have a leucine at position 254.
The sequence for the core complex layer from 0 to +8 is compiled. The +7 layer is identified with arrows. The amino acid (aa) sequence was obtained from the NIH's National Center for Biotechnology Information (NCBI; http://www.ncbi.nlm.nih.gov) and aligned using the software from DNAStar (http://www.dnastar.com).
(2.0 MB TIF)

Figure S2 Optimization for the Detection of the SDS-Resistant Complex SDS-resistant SNARE complexes isolated from 3- to 5-d-old adult fly heads are separated from syntaxin 1A monomers by SDS-PAGE and detected with an antibody (8C3) to syntaxin 1A on a Western blot (A). Syntaxin monomers and 7S SNARE complexes at different protein levels (fly heads/lane) are detected using standard ECL methods. Band intensity of the 7S complex of each lane is measured using ImageJ (NIH), normalized to the maximal intensity, and plotted against the number of fly heads loaded onto each lane (B). From three separate experiments, it appears that the optimal detection range falls between 0.25 head and one head/lane; 0.25–0.50 head was used per lane for all experiments on the 7S complex.
(6.8 MB TIF)

Figure S3 Sequencing Confirms the *syx^3–69^* Mutation in the Flies Used in Our Experiments Sequencing of the *syx^3–69^* mutant confirms the single point mutation (from ACC to ATC) resulting in a threonine to isoleucine mutation at position 254 in the *Drosophila* syntaxin 1A.
(3.3 MB TIF)

Figure S4 The Homozygous and Heterozygous *syx^3–69^* Mutants Have Similar Amounts of SNARE Complexes The amount of SDS-resistant 7S complex is similar between the homozygote and the heterozygote at 22 °C. Representative Western blots show the syntaxin 1A monomer and the 7S SNARE complex obtained from heads of the homozygote *(syx/syx)* and the heterozygote (*syx/*+ [A]). The relative level of total proteins loaded in the lanes is illustrated by the intensity of tubulin, shown at the bottom. Histograms of ratios of the 7S SNARE complex to the syntaxin monomer between these two genotypes are shown in (B). Note that the ratio is not normalized and that the difference of the ratios is not statistically significant (*p* > 0.05).
(1.5 MB TIF)

Video S1 Spontaneous Behavior at Room Temperature *Shi^ts1^, syx^3–69^, CS,* and *para^ts1^* flies (1- to 2-d-old) were mounted ventral-side up with modeling clay on a glass slide such that their legs and abdomens were allowed to move. These flies were then placed on a temperature-controlled stage under a dissection scope and movements recorded with a digital camera. Spontaneous movements of these flies were then recorded at the permissive temperature (20 °C). Note that flies from the four genotypes spontaneously extended their legs and moved their heads and abdomen.
(7.1 MB MOV)

Video S2 Spontaneous Behavior or a Lack of It at 38 °C During a 5-min period of exposure to 38 °C, the *Shi* fly (upper-left corner) and the *para* fly (lower-right corner) were completely motionless due to depletion of synaptic vesicle pools or a failure to propagate action potentials, respectively. As expected, the wild-type *(CS)* fly (lower-left corner) did not stop extending its legs or moving its head and abdomen. In contrast, the *syx^3–69^* fly constantly shook its legs and vibrated its abdomen at a high rate.
(9.2 MB MPG)

Video S3 Recovery of Spontaneous Behavior at 20 °C Upon returning to 20 °C, all four flies resumed spontaneous movements. Note that at the restrictive temperature, *Shi^ts1^* and *para^ts1^* flies were completely paralyzed, whereas the *syx^3–69^* fly constantly shook its legs and vibrated its abdomen.
(7.4 MB MOV)

Video S4 Responses in *syx^3–69^* Flies to Giant Fiber Stimulation A *syx^3–69^* (1- to 2-d-old) was mounted with its ventral side up on a slide with modeling clay and viewed with a dissection microscope at 100×. This fly was placed in a chamber whose temperature was rapidly raised to 38 °C within 45 s by a homemade temperature controller and maintained at 38 °C during the experimental period. A pair of sharp tungsten electrodes was placed into the compound eyes to electrically stimulate the giant fiber neurons in the brain (1–6 V, 100-μs duration, 5 Hz). Spontaneous and evoked leg movements of these flies were recorded using a digital camera. Note that the *syx^3–69^* fly extended its legs in response to each stimulus. Rapid and constant vibration of legs was also apparent in the *syx^3–69^* fly at 38 °C. Still clips from this video are presented in Figure 2C.
(8.9 MB MPG)

Video S5 Responses in *Shi^ts1^* Flies to Giant Fiber Stimulation Unlike the *syx^3–69^* fly, the *Shi^ts1^* fly did not respond to the electrical stimuli delivered to the giant fiber pathway when paralyzed at 38 °C. Still clips from this video are presented in Figure 2C.
(5.8 MB MOV)

## Abbreviations

*comt*: *comatose*
*CS*: *Canton-S*
DLM: dorsal longitudinal muscle
EPSP: excitatory postsynaptic potential
ERG: electroretinogram
mini: miniature synaptic potential
mEPSP: miniature excitatory postsynaptic potential
NMJ: neuromuscular junction
NSF: N-ethylmaleimide–sensitive factor
N-Syb: neuronal synaptobrevin
*para*: *paralytic*
RRP: readily releasable pool
*Shi*: *Shibire*
SNAP-25: synaptosome-associated protein-25 kDa
SNARE: soluble *N*-ethylmaleimide–sensitive factor attachment protein receptor
SV: synaptic vesicle
