# Correction: Modification of a Hydrophobic Layer by a Point Mutation in Syntaxin 1A Regulates the Rate of Synaptic Vesicle Fusion

**DOI:** 10.1371/journal.pbio.0050175

**Published:** 2007-07-17

**Authors:** Robert D Lagow, Hong Bao, Evan N Cohen, Richard W Daniels, Aleksej Zuzek, Wade H Williams, Gregory T Macleod, R. Bryan Sutton, Bing Zhang

In *PLoS Biology*, volume 5, issue 4: doi: 10.1371/journal.pbio.0050072

On page 0801, in the last sentence of the first paragraph of the Results section, “isoleucine” should be replaced with “threonine.”

The corrected version is:

“Nonetheless, the overall feature emerging from our analysis is that syntaxins with conserved threonine at the +7 layer appear to be selectively involved in regulated secretion at synapses or neurosecretory cells.”

